# Behavioural aspects of patients with Autism Spectrum Disorders (ASD) that affect their dental management

**DOI:** 10.4317/medoral.19566

**Published:** 2014-03-08

**Authors:** Jacobo Limeres-Posse, Patricia Castaño-Novoa, Maite Abeleira-Pazos, Isabel Ramos-Barbosa

**Affiliations:** 1 Grupo de Investigación en Odontología Médico-quirúrgica (OMEQUI). School of Medicine and Dentistry. Santiago de Compostela University (USC), Spain

## Abstract

Dental treatment in patients with Autism Spectrum Disorders (ASD) can be complicated due to the presence of behavioral alterations. In this group, there are no specific behavioral profiles that allow dentist to anticipate the attitude that a patient will show during a visit. Thus, behavioral attitudes have been described that vary from total permissiveness and collaboration during even bloody procedures, to the absolute impossibility in conducting a simple oral examination. There is no effective behavioral management technique for all ASD patients. Prior information, such as the type of ASD or the presence of certain concurrent pathologies can help predict the patient’s likely behavior. Therefore, gathering all the information in a preliminary interview with the parents/guardians of the patient is recommended. Knowing these factors will allow individualized behavioral management strategies to be designed and facilitates the planning of dental treatment.

** Key words:**Dentistry, autism, ASD, behavior management.

## General characteristics of autism spectrum disorders

Autism is defined as an alteration in neurodevelopment characterized by severe damage in social interaction, language, behavior and cognitive function (1,2). The distinct patterns of the illness are unified in a classification system referred to as Autism Spectrum Disorders (ASD) (1,2), traditionally subdivided into 3 large groups. Classic autism (or “autism disorder”) is the most severe profile and presents severe alterations on the cognitive, social, and behavioral levels. ‘Pervasive development disorder not otherwise specified’, is a diagnosis reached by including patients with similar problems to autism, but which do not reach the diagnostic criteria in number, severity, or age of presentation (3), and tend to present more activity, social interaction, and empathy than classical autistics (1,3). Asperger syndrome is characterized by a relatively normal language and intellect quotient, but with a deficit in social abilities, a reduction in the ability to show empathy, and often an unusual interest in something (1-3).

Towards the beginning of 1990s, it was estimated that ASD affected 1 in 1,500 live births. Nevertheless, Rice *et al*. (4) published a study in 2004 in which this prevalence rose notably, to 1 in 150 births. More recent data, provided by the Centers for Disease Control and Prevention (Atlanta, USA), estimate that for those under 8 years of age, 1 in 110 children suffer some form of ASD, with a man-woman ratio of 3.7:1.0 (5,6). Many factors have been identified that could justify this increase in ASD prevalence such as: more information and greater interest on the part of parents, the modification of diagnostic criteria, and better training as regards ASD amongst health and education professionals. Suggesting that as a consequence of all these factors, there could currently be a certain amount of overestimation (7).

The etiology of ASD is still unknown, although in some patients structural and functional alterations in the limbic system (responsible for emotions and social relations) have been detected through use of sophisticated imaging techniques (8). The selective hypo-activation of certain cerebral areas associated with motor integration has also been described (9). In studies carried out on affected families, an inherited disorder has been described that affects 20 genes located on chromosomes 2,7,15,16 and 19 that interact amongst themselves (10). The predominance amongst males suggests an alteration associated to the Y chromosome, but paradoxically, studies of the genome of patients with ASD relate it to the X chromosome (10).

Intellectual disability is frequent in people with ASD, having an estimated prevalence of 75% (1,2). Verbal and non-verbal communication tends to be limited or nonexistent (50% of patients do not acquire spoken language). Their language is often repetitive and does not correspond to a context, demonstrating lack of comprehension (11). Immediate or delayed echolalia is present in 75% of children (11,12). Their interests and activities tend to be limited, and repetitive behaviors are frequent, often set off by stress, excitement, or certain stimuli (such as noises) (1,13). They tend to acquire strict routines to which changes or modifications can give rise to resistant attitudes (1,2,13). They may also present a lack of motor coordination and repetitive body movements (1,2,13,14). One of the principal characteristics of children with ASD is their low frustration threshold, which frequently leads to tantrums. As they grow older, agitation, aggressiveness, and self-wounding can appear. Furthermore, they may suffer concurrent mental disorders such as anxiety, mood swings, attention deficit and hyperactivity, obsessive-compulsive disorder, or schizophrenia (which is more prevalent during adolescence) (2,14,15). Sensory perception may also be affected with frequent showing of auditory and tactile hypersensitivity, exaggerated reactions to light and smells1, an inadequate response by the vestibulovisual system with predominant focal over peripheral vision, and an elevated pain threshold (1,14,16,17).

The diagnosis of ASD is established after a careful medical, psychological, and neurological examination, and is based on 4 criteria (1-3): serious alterations in social relations; serious alterations in the development of communication; patterns of behavior, interests and activities that are restricted, repetitive, and stereotyped; and early onset (before 3-5 years of age). Not all of these symptoms must necessarily be present simultaneously or with the same intensity. One of the main problems in achieving a definitive diagnosis is rooted in the lack of genetic, medical, or specific analytical tests (1,2).

Currently, one of the most commonly used treatments is encompassed under the pedagogic concept named “TEACCH” (*Treatment and Education of Autistic and Communication related handicapped Children*) (18) that unites special education, behavior management, language therapy, and social training techniques. One of the essential tools of TEACCH is visual learning (18). Pharmacological treatment does not have any effect on the ability to socialize or communicate, and is reserved for the control of some symptoms associated with ASD, such as epilepsy, anxiety, or irritability (19). The most commonly used pharmaceuticals are: risperidone, olanzapine, fluoxetine, sertraline, carbamazepine, valproic acid, clonidine, and methylphenidate (3,19).

## The state of oral health of patients with asd

Patients with ASD do not present any specific dental characteristic in the soft or hard intraor perioral tissues (14,17,20,21). Although ASD is associated to certain determinants of oral health (institutionalization, diminished motor skills, the swallowing whole of food, bruxism, the administration of pharmaceuticals, etc.), the majority of studies report low indices of caries in this group (17,20), or at least comparable levels to the non-disabled population (21,22). Some authors even reported a lower prevalence of caries in ASD patients when compared to healthy control groups (14,23-25). Bruxism is present in between 20 and 60% (14,25). Gingivo-periodontal pathology is more prevalent in patients with ASD compared to healthy control groups (21,22,24). These differences are explained by the poorer levels of oral hygiene seen in ASD patients (22,24). Therefore, some authors highlight periodontal treatment, oral hygiene techniques, and nutrition as amongst the main dental treatment needs of this group (17). The presence of adverse effects on the oral cavity from medicines have also been described, particularly hyposalivation (paroxetine, fluoxetine, imipramine), oral ulcers (carbamazepine), delayed scarring (valproic acid) or gingival enlargement (phenytoin).

## Behaviour management of the asd patient in dentistry

**Figure 1:**
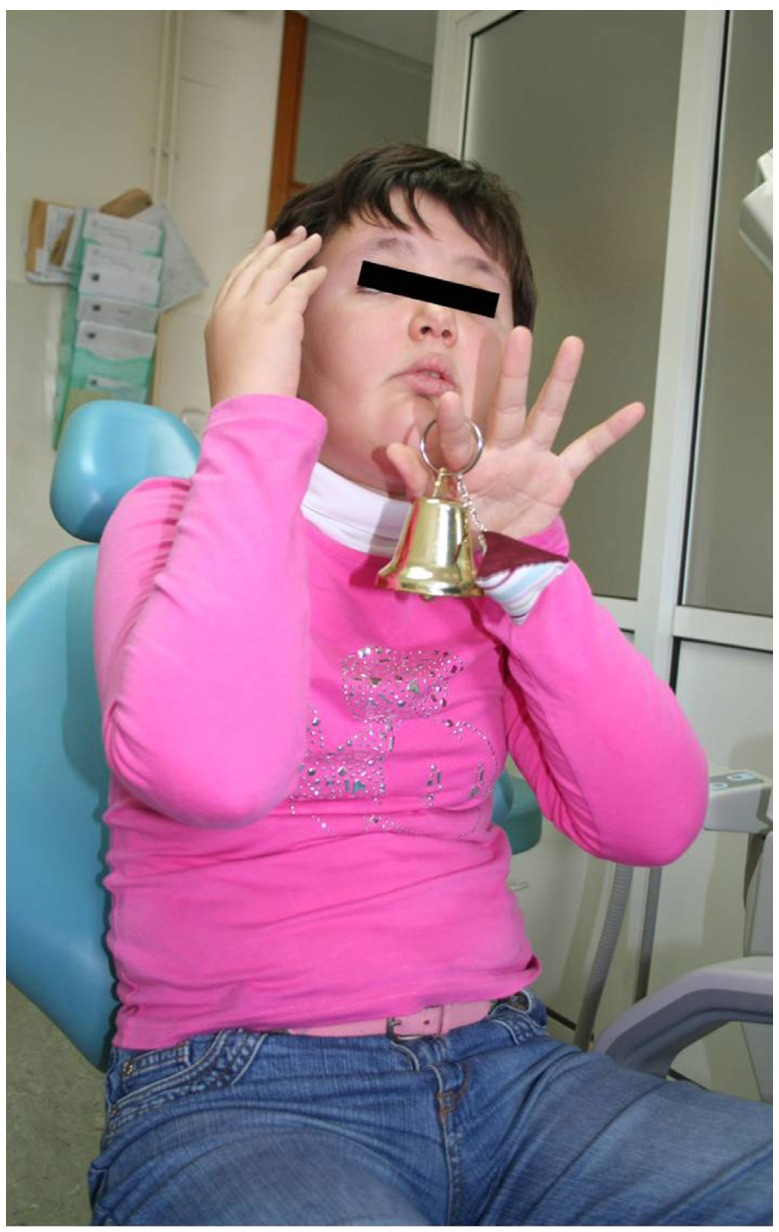
An attitude of avoidance of a patient diagnosed with ASD in a dental surgery.

People with ASD may be unable to cooperate in the dental clinic due to their difficulties with social interaction and communication (Fig. 1) (3,17). Moreover, cognitive dysfunction, the presence of aggressiveness, convulsions and other associated symptoms, reduce the possibility of being treated on an outpatient basis (13). Resistance to changes in routine limits the ASD patient in developing a positive attitude in the dental clinic (26,27). Many authors have analyzed what factors are related to the behavior of these patients in dentistry. Loo *et al*. (26) highlighted three characteristics which should always be taken into account:

-The age of the patient: an increase of 1 year is associated with an 8% reduction in the probability of the manifestation of disruptive behaviour. Therefore, the older a patient, the higher his or her cooperation.

-Associated pathologies: patients diagnosed with intellectual disability, cerebral paralysis, self-mutilation, or Pica disorder have a higher probability of poor cooperation as opposed to patients without these associated pathologies.

-Diagnosis of ASD: patients diagnosed with autism disorder are significantly less cooperative than those diagnosed with Asperger’s syndrome and Pervasive Development Disorder.

In the same study, other variables such as sex, CAOD, primary residence, epilepsy, psychotropic medication, and prior history of rehabilitation or surgical treatment were also analyzed in relation to behavior at the clinic. None of these variables were statistically related to the type of behavior shown during the dental consultation (26).

Marshall *et al*. (27) studied 26 possible determining factors in the level of cooperation of patients with ASD when faced with dental treatment. They obtained five independent variables considered to be risk factors for non-cooperation: age (4-7 years vs. >7 years), ability to read (no vs. yes), sphincter control (no vs. yes), associated systemic diagnosis (yes vs. no) and ability to speak (no vs. yes). Presenting 2 or more risk factors was shown to be closely related to non-cooperative behavior. Furthermore, 65% of patients with ASD were uncooperative during the dental treatment sessions. In light of these studies, some authors consider useful to interview parents/guardians in a preliminary visit in order to have information regarding these variables, with the goal of identifying the potentially cooperative patient and designing behavior management strategies (26-28).

It has been demonstrated that some patients with ASD can be “trained” to tolerate dental procedures (29). Nevertheless, in addition to the characteristics of each patient, success is determined by constraints of time, money, and human resources (28). There is no one system applicable to all patients diagnosed with ASD. Of the most frequently used, the following may be highlighted:

-Visual Pedagogy

**Figure 2:**
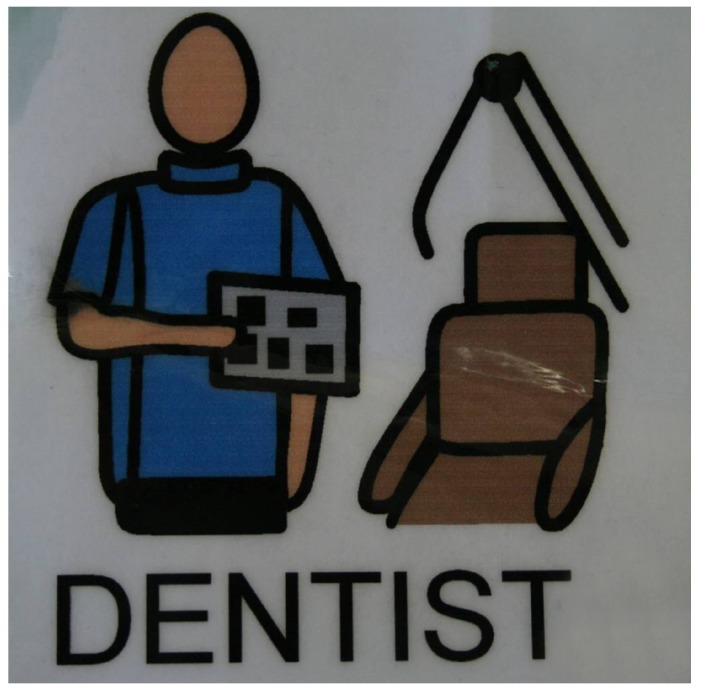
Pictogram designed for the familiarization of the ASD patient with the dental environment.

One of the basic principles of TEACCH is the structuring of time and space, given that patients with ASD react more favourably to structured, as opposed to unstructured, situations (18). This structuring is carried out through drawings (pictograms, photos, etc.) that systematically describe all the aspects of life, from the routine to sporadic events (Fig. 2). Through visual pedagogy, what to do, where, when, and how are explained, as well as what to do after (18,29,30). Bäckman and Pilebro (29) evaluated a model based on visual teaching to introduce dentistry to preschool children with autism disorder, resulting in an effective method that notably increased their ability to cooperate. The same authors carried out a prospective study in 2005 on 14 autistic patients in order to apply visual pedagogy to tooth brushing. After 12 months of follow-up, a significant reduction in visible dental plaque was achieved, and at 18 months, the majority of the parents affirmed that the carrying out of the oral hygiene techniques was easier than at the beginning of the study (30).

-Behavioural Techniques

There are general guidelines applicable to all patients with ASD, such as the importance of getting as much prior information as possible from the parents/guardians (routines, fixations, fears, etc.) (17). Furthermore, on occasion their presence in the clinic can be useful, increasing trust and level of cooperation. Short visits are recommended, and sensory stimuli (noises, smells, etc.) should be reduced to the minimum. Patients with ASD get distracted easily, therefore as few movements as possible must be made while working, and touching the patient’s sides should be avoided whenever possible. If the patient exhibits inappropriate behaviour, it is recommended to be ignored from the beginnig (14,17,26).

In addition to these general measures, there are varying behavior management techniques that can be useful during the dental treatment of patients with ASD. Normally it is necessary to use more than one for each patient during the course of treatment (20).

-Communication Techniques

Say-show-do, in spite of its great effectiveness in pediatric patients, has been catalogued as poorly effective in patients with ASD because of their limited attention span (28). Patients often do not respond to the demonstrations, and they are resistant to establishing personal contact, which results in difficulty in introducing procedures and dental instruments (31). Voice control accompanied by facial expression is also a typical technique in children. Nevertheless, difficulty understanding language and interpreting emotions have been shown as factors that reduce their use in patients with ASD. In order to increase the chances of success, orders must be clear, short, and employ simple sentences that the patient knows (17,28).

-Behaviour modification techniques

Desensitization consists of a first contact with the dental sphere before the carrying out of the dental procedure (20). Appointments must be as repetitive as possible, scheduling them the same day of the week, at the same time, with the same personnel, in the same dental chair, minimizing waiting time and the total time spent in the clinic. It has been suggested that the patient practice at home and/or in his or her education centre, getting familiar with the instruments and procedures, including orders that will later be dictated by the dentist such as “hands down” or “look at me” (17). It is a progressive approach that takes a lot of time and for which it is impossible to foretell which patients will have success or the number of sessions that may be necessary. Positive reinforcement is also a therapy with reduced efficacy. Limitations in receptive ability and attention span result in the reinforcement exceeding the emotional alteration derived from the dental consultation. Furthermore, classic “reinforcement objects” can lack value for the patient with ASD, and tend to be given at the end of the consultation and at the moment the desired behavior is produced, which reduces their impact. Therefore, it can be useful to know if the patient has some sort of “rescue” object to which he or she goes in stressful or anxious situations. In such a case, it is recommended to have the object in the clinic during treatment ready to use if necessary.

-Physical focus techniques

Bite blocks or mouth openers can help achieve a mouth opening that is sufficient to carry out dental treatment and maintain the bite reflex under control. Their use is highly variable in literature, but some authors have still highlighted their usefulness in patients with ASD (13). Physical restraint is a controversial technique. Kamen and Skier (14) pointed out that its use was unnecessary and ineffective in the management of problematic behavior in patients with ASD. Still, authors such as Klein (17) and Lindemann (32) note that by applying restraint devices they achieved a “calming” effect on patients. In spite of the existing controversy, the use of physical restraint is recommended to avoid possible aggressive or self-mutilating behavior (17,32).

In spite of these techniques, in many situations the dental treatment will require the use of pharmacological techniques for behavioral management.

-Pharmacological behavior management techniques

Before prescribing a medicine for conscious sedation, it is recommended that a medical history is available (especially concurrent pathologies and medications) and that information is obtained regarding previous uses of this sort of pharmaceutical agent (17). In this group, atypical reaction patterns often appear, and frequently, the standard doses and pharmaceuticals are ineffective. Various sedative agents used alone or in combination have been suggested (31,33). Among the most commonly referred to are: nitrous oxide; diazepam; hydroxyzine chlorhydrate; alphaprodine chlorhydrate; prometazine hydrochloride; and chloral hydrate, but although they are still common in the literature, their success is limited (14,31,33). Davila and Jensen (31) used the pharmaceuticals previously mentioned alone and in combination, in differing doses, over a period of 10 years on the same patient. In spite of the multiple combinations, they were not able to develop a predictable sedation protocol. In relation to nitrous oxide, its administration requires a certain level of communication with the patient, which can be difficult to achieve in patients with ASD. It has been pointed out that a slower administration and higher concentrations are necessary as compared to healthy population in order to achieve an adequate level of sedation (33) Performing dental treatment under General Anesthesia (GA) should be considered when the patient does not respond to the techniques mentioned (31).

The behavior exhibited in the clinic by the patient with ASD tends to be the determining variable in the decision whether or not to opt for GA. Nevertheless, the need to carry out extensive (4 quadrants of the oral cavity affected) and/or complex (prosthetic rehabilitation) treatments has also been noted as a factor associated with the use of GA. The percentage of patients with ASD that require treatment under GA varies between 37% and 76% (20,26,34).

Different authors have analyzed which of the aforementioned strategies is the most adequate for behavior management in patients with ASD in the dental clinic. Communicative behavior modification techniques alone or in combination are frequently used. Klein and Nowak (20) affirmed that communicative techniques complemented with differing levels of restraint constitute the first option that often proves successful. Still, in their series they reported that in 37% of their patients it was ineffective, having to resort to treatment under GA in these cases. Watanabe *et al*. (35) proposed the combination of desensitizing techniques along with nitrous oxide sedation. In their study, it was effective in 87.5% of the patients (average age, 11 years), who did not need physical restraint in order to undergo treatment but who had needed it for prior treatments. In the case of more complicated patients (worse behavioral alterations, aggressiveness, etc.), the most recommended methods are physical restraint, conscious sedation with orally administered midazolam (whether associated or not with nitrous oxide) and GA (26) Authors such as Klein and Nowak (20) refer to a greater use of physical restraint than GA, as opposed to others such as Loo (26) and Kamen and Skier (14), who in most cases turn to the latter.

In conclusion, working with patients with ASD in the dental clinic is still a challenge for the professional. There is no protocol for behavior management applicable to all patients. Information such as the type of ASD or the presence of certain concurrent pathologies can orient one to the patient’s behavior, therefore it is recommend that this information is gathered in a preliminary interview with the parents/guardians of the patient. With this information, adaptation strategies ought to be designed for transition to the dental environment, remembering that a considerable percentage of patients will require treatment under GA.
